# Simultaneous Treatment of Complex Incisional Hernia and Stoma Reversal

**DOI:** 10.3389/jaws.2023.11093

**Published:** 2023-01-27

**Authors:** Carles Olona, Ricard Sales, Aleidis Caro-Tarragó, Beatriz Espina, Raquel Casanova, Rosa Jorba

**Affiliations:** Hospital Universitari Joan XXIII de Tarragona, Tarragona, Spain

**Keywords:** incisional hernia, simultaneous surgery, ostomy reversal, ostomal hernia, complex incisional hernia

## Abstract

**Purpose:** The simultaneous repair of incisional hernias (IH) and the reconstruction of the intestinal transit may pose a challenge for many surgeons. Collaboration between units specialized in abdominal wall and colorectal surgery can favor simultaneous treatment.

**Methods:** Descriptive study of patients undergoing simultaneous surgery of complex IH repair and intestinal transit reconstruction from the start of treatment in a joint team. All interventions were performed electively and with the collaboration of surgeons experts in abdominal wall and colorectal surgery.

**Results:** 23 patients are included. 11 end colostomies, 1 loop colostomy, 6 end ileostomies and 5 loop ileostomies. Seven (30%) patients presented with a medial laparotomy incisional hernia, 3 (13%) with a parastomal incisional hernia, and 13 (56%) with a medial and parastomal incisional hernia. Closure of the hernial defect was achieved in 100% of cases, and reconstruction of the intestinal tract was achieved in 22 (95%). Component separation was required in 17 patients (74%), which were 11 (48%) posterior and 6 (26%) anterior. In-hospital morbidity was 9%, and only two patients presented Clavien-Dindo morbidity > III when requiring reoperation, one due to hemorrhage of the surgical bed and another due to dehiscence of the coloproctostomy. The mean follow-up was 11 months, with 20 (87%) patients having no complications. Mesh had to be removed in one patient with anastomotic dehiscence, no mesh had to be removed due to surgical site infection.

## Introduction

The surgery for complex abdominal incisional hernia is a challenge for surgeons who are not specialized on the abdominal wall, just as the reconstruction of the intestinal tract in patients with ostomies can be a challenge for non-expert surgeons.

Patients with some type of temporary ostomy mostly have an incisional hernia at the time of ostomy closure, which may make associated morbidity more likely (1). In many cases, the treatment of only one of the pathologies is opted for, leaving the patient without definitive treatment, with the possibility that their symptoms persist and their quality of life worsens. Although there are no recommendations for a standardized treatment due to the absence of high-level scientific evidence, publications from specialized centres have recently appeared in which the possibility of the simultaneous approach to these pathologies is described (2, 3).

Previously, in our department, these procedures were performed by several surgeons, in many performing only the ostomy reversal and postponing the treatment or, in many cases, leaving the incisional hernia untreated, and their evolution was not recorded either.

With the hypothesis that the simultaneous approach to the ostomy reversal and incisional hernia by a team of surgeons specialized in colorectal surgery and abdominal wall surgery allows its treatment safely and in a single operation, we created a specific management protocol and surgical team.

This article describe our method of the simultaneous approach and analyze the results obtained.

## Materials and Methods

The study is carried out in a University Hospital, reference center in the province for colorectal and complex abdominal wall surgery. In 2019 we established a team made up of surgeons specialized in colorectal surgery and surgeons with extensive experience in abdominal wall techniques, for the individualized treatment of patients who require ostomy reversal and abdominal wall reconstruction.

### Therapeutic Strategy

All patients were initially evaluated by colorectal surgeons who assessed the intestinal state by colonoscopy and contrast enema and established the indication of intestinal tract reconstruction.

The abdominal wall was assessed by abdominal CT scan with the determination of the diameter of the incisional hernia and the abdominal and hernia volumes to determine the Tanaka index (4) to estimate the risk of loss of domain of the hernial content.

Each case was evaluated jointly by colorectal surgeons, abdominal wall surgeons, and stoma therapists, who chose the specific surgical technique based on the conditions and requirements of each patient. Patients were informed of the decided procedures and their possible complications and signed the corresponding informed consent. They followed a prehabilitation programme, after which they were considered suitable for surgical treatment if they had stabilization of their cardiopulmonary disease, absence of tobacco consumption, body mass index (BMI) <35, and, in cases of diabetes mellitus, HbA1c <8.

At the same time they were evaluated, prehabilitation of the abdominal wall was performed with botulinum toxin A in patients with incisional hernias with a transverse diameter greater than 12 cm, and/or preoperative progressive pneumoperitoneum if considered necessary for loss of domain, following the criteria published by Ibarra-Hurtado (5) and Bueno-Lledó (6).

The surgical intervention was scheduled jointly with surgeons from the colorectal pathology unit and the abdominal wall unit. Patients received mechanical bowel preparation and oral antibiotic therapy according to the protocol of the colorectal surgery unit. In the induction of anesthesia, intravenous antibiotic prophylaxis was administered. The recommendations for the prevention of surgical site infection of the Spanish Association of Surgeons (7) and the Surveillance Program for Nosocomial Infections in hospitals of Catalonia (VINCAT) measures were followed. The initial approach was performed by midline laparotomy, the incisional hernia sac was dissected, and the abdominal adhesions were released. The ostomy was isolated, and the hernia sac was dissected if present. Ileoileostomy was performed in cases of loop ileostomy, ileoproctostomy in cases of subtotal colectomy, and mechanical coloproctostomy in cases of Hartmann reconstruction.

The technique of choice for the repair of the incisional hernia was retromuscular, along with posterior component separation (Transversus Abdominis Release, TAR) in hernias with a transverse diameter up to 10 cm. In cases with transverse diameter greater than 10 cm o with impossibility of retromuscular repair, an anterior components separation was performed. The midline was closed by a short-stitch technique with 2/0 polydioxanone slowly absorbable suture. The type of mesh used was medium-weight macroporous monofilament polypropylene (Optilene, BBraun, Barcelona, Spain) or polyvinylidene fluoride (Dynamesh CICAT, FEG Textiltechnik, Aachen, Germany) according to the choice of the specialist surgeon. When Transversus Abdominis Release (TAR) was performed a second biosynthetic mesh (Bio A, WL Gore & Associates Inc., Bozeman, United States) was aded, In cases with excess adipose-cutaneous tissue, an associated panniculectomy was performed. Drains were placed in cases with large subcutaneous dissections or the placement of onlay mesh, and removed when their debit was less than 50 mL/24 h.

### Statistical Analysis

All patients with some type of ostomy who required intervention for the reconstruction of the intestinal tract and repair of a complex incisional hernia from January 2019 to December 2021 were included. All data were collected prospectively in the National Registry of Incisional Hernia (Registro Nacional de Hernia Incisional) (8), promoted by the Abdominal Wall Section of the Spanish Association of Surgeons (Asociación Española de Cirujanos) and approved by the Ethics Committee of Scientific Research of the centre. The data were stored on an external server so that each centre could access their data in a private and confidential way.

Demographic data such as age, sex, body mass index, comorbidities, and toxic habits were collected. Data related to the incisional hernia, such as size, classification according to the European Hernia Society (EHS), association with incisional parastomal hernia, and degree of wound contamination according to the US Centers for Disease Control and Prevention (CDC) (9). Characteristics of the intervention, such as its association with intestinal resections, the separation of components and their type, the number and type of mesh placed, and the surgical technique performed. The main follow-up outcome was the presence of a surgical site infection and surgical site occurrence defined as other wound events that are not captured by SSI (seroma, wound dehiscence, hematoma, enterocutaneous fistula) (10). If SSI is present, its CDC classification (superficial incisional, deep incisional, or organ-space SSI). Other postoperative complications were the presence of intestinal or cutaneous dehiscence, and recurrence. In the long-term follow-up, the degree of success, data on relapses, and data on reoperations are determined.

The data are detailed through descriptive analysis. The qualitative variables are expressed as absolute numbers and percentages, and the quantitative variables as mean and range.

## Results

During the study period, 23 patients underwent simultaneous repair of complex incisional hernia and intestinal tract reconstruction. They were nine women and 14 men with a mean age of 63 years. The demographic variables and previous pathology are detailed in Table 1. The mean body mass index (BMI) was 28.5 kg/m^2^ (21–34). Seven patients were diabetic (30%), and 4 (17%) were smokers. According to the classification of the American Society of Anesthesiologists (ASA), one patient (4%) was classified as ASA I, 12 (52%) as ASA II, and 10 (43%) as ASA III. They had had a mean of 2.2 (0–5) laparotomies before the reconstruction surgery, We consider 0 laparotomies if the surgery that originates the colostomy has been performed laparoscopically.

**TABLE 1:** Patients characteristics.

Variable	*n* = 23
Gender	
Male	14 (60%)
Female	9 (39%)
Age [years]	63 (40–85)
Body mass index	28.5 (21–34)
Diabetes	7 (30%)
Heart Disease	5 (22%)
Chronic obstructive pulmonay disease	3 (13%)
Malignant neoplasm	12 (52%)
Smoking	4 (17%)
Alcohol	4 (17%)
ASA classification	
I	1 (4%)
II	12 (52%)
III	10 (43%)

Numbers are mean (range) or N (%).

The previous surgeries in which the ostomy was performed are detailed in Table 2. They were 11 (48%) terminal colostomies, 1 (4%) lateral colostomy, 6 (26%) terminal ileostomies, and 5 (22%) lateral ileostomies.

**TABLE 2:** Previous surgeries in which the ostomy was performed.

Type of ostomy	*n* = 23
End colostomy	**11**
Anastomotic dehiscence	4
Ischemic colon perforation	1
Polytrauma	2
Stenosing neoplasm of rectum	1
Stenosing distal anastomosis	1
Diverticular peritonitis	2
Loop colostomy	**1**
Occlusive neoplasm	**1**
End ileostomy	**6**
Mesenteric ischemia	2
Anastomotic dehiscence	4
Loop ileostomy	**5**
Anterior resection of rectum	3
Anastomotic dehiscence	2

Bold values are subtotals by type of ostomy.

Table 3 describes the characteristics of the hernias. The mean transverse hernial diameter was 11.9 cm (4–20), and the longitudinal diameter was 17.4 cm (4–40). The average time elapsed since the previous intervention was 2 years. Seven (30%) patients presented with a midline laparotomy incisional hernia, 3 (13%) with a parastomal incisional hernia, and 13 (56%) with a midline and parastomal incisional hernia. Previous treatment with botulinum toxin A was required in 7 (30%) cases.

**TABLE 3:** Hernia characteristics.

	Total N = 23	Terminal colostomy N = 11	Lateral colostomy N = 1	Terminal ileostomy N = 6	Lateral ileostomy N = 5
Hernia type					
Medial	7 (30%)	2 (18%)	0	5 (83%)	0 (0%)
Parastomal	3 (13%)	2 (18%)	0	0 (0%)	1 (20%)
Medial + Parastomal	13 (56%)	7 (63%)	1	1 (17%)	4 (80%)
Hernia width, cm	11.9 (4–20)	12.7 (4–20)	16	10.6 (6–14)	11.6 (6–15)
Hernia length, cm	17.4 (4–40)	16.1 (4–40)	19	22.5 (15–30)	13.8 (6–25)
Reduction capacity					
Totally	14 (61%)	8 (72%)	1	2 (33%)	3 (60%)
Partially	9 (39%)	3 (27%)	0	4 (66%)	2 (40%)
Waiting list [years]	2 (0.5–7)	1.85 (1–7)	1	2.1 (0.5–3)	2.2 (1–4)
Number of prior laparotomies	2.2 (0–5)	2 (0–4)	1	2.7 (1–5)	2.7 (1–5)
Recurrent hernia	1 (4%)	0	0	0	1 (20%)

Numbers are mean (range) or N %.

Table 4 details the characteristics of the surgical interventions. Closure of the hernial defect was achieved in 100% of cases, and reconstruction of the intestinal tract was achieved in 22 (95%). The average duration of surgery was 200 min (65–382). Colon resection was performed in 16 (69%) cases, and small intestine resection was performed in 5 (22%) cases. Component separation was required in 17 patients (74%), which were 11 (48%) posterior and 6 (26%) anterior. Out of all the cases, 18 (78%) retromuscular, 4 (8%) onlay, 1 (4%) preperitoneal, and 6 (26%) double meshes were placed. In 4 (17%) cases, panniculectomy was also done.

**TABLE 4:** Characteristics of surgical interventions.

	Total N = 23	Terminal colostomy N = 11	Lateral colostomy N = 1	Terminal ileostomy N = 6	Lateral ileostomy N = 5
CDC wound class					
Clean-contaminated	11(48%)	6(54%)	0	2(33%)	3(60%)
Contaminated	12 (52%)	5 (45%)	1	4 (66%)	2 (40%)
Fascial closure	23 (100%)	11 (100%)	1	6 (100%)	5 (100%)
Ostomy reversal	22 (95%)	10 (91%)	1	6 (100%)	5 (100%)
Operative time, min	200 (65–382)	210 (89–382)	382	196 (140–240)	169.5 (65–240)
Preoperative Botulinum Toxin	7 (30%)	3 (27%)	0	3 (50%)	1 (20%)
Panniculectomy	4 (17%)	2 (18%)	0	1 (16%)	1 (20%)
Bowel resection	21 (91%)	11 (100%)	1	5 (83%)	4 (80%)
Colonic resection	16 (69%)	8 (73%)	1	3 (50%)	4 (80%)
Small bowel resection	5 (22%)	3 (27%)	0	2 (33%)	0
Surgical Technique					
Onlay	2(4%)	2(18%)	0	0	0
Retrorectal	4(8%)	1(9%)	0	1(16%)	2(40%)
Anterior Component Separation	6(26%)	2(18%)	0	3(50%)	1(20%)
Posterior Component Separation	11(48%)	6(54%)	1	2(33%)	2(40%)
Mesh	23 (100%)	11 (100%)	1	6 (100%)	5 (100%)
Single mesh	17 (74%)	8 (73%)	1	3 (50%)	5 (100%)
Double mesh	6 (26%)	3 (27%)	0	3 (50%)	0
Mesh width, cm	23.4 (15–45)	21.6 (15–45)	30	22.5 (15–30)	22.5 (15–30)
Mesh length, cm	25.8 (13–45)	24.3 (13–45)	30	26.6 (20–30)	25 (15–30)
Mesh position					
Onlay	4 (8%)	2 (18%)	0	1 (16%)	1 (20%)
Retromuscular	18 (78%)	9 (82%)	1	5 (83%)	3 (60%)
Preperitoneal	1 (4%)	0	0	0	1 (20%)

Numbers are mean (range) or N %.

The average stay was 10.4 days. In-hospital morbidity was 9%, and only two patients presented Clavien-Dindo morbidity > III when requiring reoperation. The first patient underwent reoperation due to retrorectal bleeding, the previous mesh was removed, hemostasis and closure were performed with the placement of a new retrorectal mesh. The second case presented dehiscence of the colorectal anastomosis, the mesh was removed and an end colostomy was performed. Closure of the abdominal wall was not possible due to abdominal hypertension, it was treated with open abdomen and mesh mediated closure with negative pressure therapy (NPT). Definitive abdominal wall reconstruction with double mesh by the abdominal wall team was performed after 6 NPT replacements.

The mean follow-up was 11 months, with 20 (87%) patients having no complications. There were 4 (17.3%) surgical site infections, 2 (9%) superficial infections treated with wound opening, 1 (4%) deep infection treated with negative-pressure therapy, and 1 (4%) organ-space infection treated by anastomotic dehiscence. 3 SSI at 30 days and 1 detected at 90 days control. No mesh had to be removed due to surgical site infection, neither late mesh infection was detected (Table 5).

**TABLE 5:** Postoperative morbidity at 30 and 90 days.

N = 16	N (%)
Intraoperative complications	0
Average length of stay (days)	10.4 (3–64)
30 days SSO (including SSI)	9 (39%)
30 days SSI	3(13%)
Wound opening	1 (4%)
Bleeding	1 (4%)
Seroma	2 (8%)
Hematoma	2 (8%)
90 days SSI	1 (4%)
Unplanned reoperations	3 (13%)
Wound bleeding	1 (4%)
Intestinal obstruction	1 (4%)
Anastomotic dehiscence	1 (4%)
Mortality	0

SSO, surgical site occurrence; SSI, surgical site infection. Numbers are mean (range) or N %

## Discussion

At the time of performing intestinal reconstruction in patients with some type of temporary ostomy, more than 22% of them have an incisional hernia of the previous laparotomy, which may also be associated with a parastomal incisional hernia (11). In high-risk patients, the incidence can reach 40% (12). The simultaneous surgical treatment of both pathologies has the advantage of avoiding additional interventions, but a higher risk of complications has also been described due to the longer surgical time and risk of surgical site infection.

The absence of a unified criterion for its treatment, along with the traditional reluctance to use meshes in contaminated fields, has led to the choice of two-stage treatment in many cases for this pathology (13). This two-stage treatment is also not free of complications, since the dissection of planes and adhesiolysis performed for stoma reversal can damage the fascial quality and complicate the later repair of the abdominal wall (14). On the other hand, the appearance of any complication in the postoperative period of the first intervention can prevent the definitive repair of the pathology, with the consequent deterioration of the quality of life of the patient and even urgent hernia complications that require palliative treatment with high morbidity and mortality, as well as an increased risk of recurrence.

The recent appearance of complex abdominal wall repair techniques that allow the reconstruction of the abdominal wall (2), the specialization of surgeons in complex wall surgery, the safety of the use of synthetic meshes in contaminated fields (14), and the better preoperative optimization of patients (3) have allowed certain groups to decide on the simultaneous treatment of this type of pathology.

The extreme variability in this type of patient makes it practically impossible to perform randomized prospective studies. Many of the studies that compare reconstruction in one or two stages only compare simultaneous performance with ostomy reversal without reconstruction of the abdominal wall (13, 15). Therefore, we think that the groups are not comparable. In our series, the hernia diameters at the time of the intervention exceeded 10 cm on average, so they required component separation techniques for their correct repair (74% of the intervened cases). These are techniques that, due to their complexity, can lead certain non-expert surgeons to refuse to repair the hernia and opting to perform stoma closure only.

We propose a protocol for this simultaneous procedure that includes the collaboration of surgeons who are experts in both colorectal and abdominal wall pathologies, which minimizes all the factors that influence the appearance of morbidity in this type of patient. The selection of patients and their prehabilitation results in a patient group with a mean BMI lower than 30, to reduce the morbidity associated with obesity. Similarly, we have encouraged patients to stop smoking.

All interventions were classified into contaminated or clean-contaminated groups, with intestinal resections in 91% of cases. In one case, stoma reversal could not be performed due to the extensive adhesion that prevented the correct dissection of the rectal stump.

We included in the prehabilitation the injection of botulinum toxin in patients with transverse hernia diameters greater than 12 cm, which we performed in 30% of cases. We believe that this prehabilitation allowed us to obtain complete fascial closure in 100% of the cases. The retromuscular position of the mesh, performed in 78% of cases, allows greater resistance to infection due to better irrigation and decreased risk of seroma with respect to the onlay position. We opted for onlay placement in large hernias in which it was only possible to perform an anterior separation of components.

One of the factors classically considered for the realization of these reconstructions in two stages is the risk of surgical site infection when using a mesh in CDC grade II-III surgery (16). However, recent studies show that monofilament macroporous synthetic meshes, such as those we used, do not cause a worsening of the postoperative course of the surgical wound (17) and are even more cost-effective than biological treatments in clean-contaminated wounds (18). The recommendation is made based on the large amount of surgical evidence of the use of permanent synthetic meshes in grade II-III fields (19). The figures on surgical site occurrences at 30 postoperative days that we have obtained are comparable to those existing in the current literature (30%) (14). We highlight that only 3 (13%) patients presented surgical site infection without requiring surgical reoperation or removal of the mesh in any case. There were three reoperations, one for postoperative hemorrhage, not related to the use of prostheses, another for anastomotic dehiscence, and a third in the late postoperative period for intestinal occlusion due to adhesions, not related to previous surgery. The low long-term morbidity, mainly surgical site infection, allows us to conclude that the use of this type of mesh, always associated with all surgical infection prophylaxis manoeuvres, allows the simultaneous performance of these procedures.

We recognize that this study has some limitations. First, it is a short series of cases, all from a single centre, although this fact also allowed us to limit the conditions in which the procedure was performed, such as the same surgical team with the collaboration of expert surgeons in each field. Another limitation is that it is a retrospective study, although it is based on a prospectively collected database. The complexity of the cases makes it difficult to standardize the surgery and to perform a randomized prospective study and this is why we consider that these patients requires to be managed with the collaboration between surgical teams. The diversity of surgical techniques used has forced us to carry out a descriptive study, since it is not possible to make any type of comparison with sufficient significance. Comparison with a control group could not be made because abdominal wall surgery had not been treated or registered in patients undergoing stomal reversal.

## Conclusion

The lack of large or prospective studies does not allow us to determine which is the best treatment for patients with complex incisional hernias that also require intestinal tract reconstruction. The complexity of the cases makes a correct study and prehabilitation of the patients essential.

The technical advances and the improvement of prosthetic materials, together with the specialization in colorectal and abdominal wall surgery, allow that with the collaboration between surgical teams, these two pathologies can be treated simultaneously in a single session, allowing a rapid physical recovery with the consequent improvement of their quality of life, reduction of surgical waiting time, and possible economic savings.

Larger and prospective studies needed to determine the best treatment for these complex cases.

## Data Availability

The raw data supporting the conclusion of this article will be made available by the authors, without undue reservation.
